# Feasibility of Performing Multiple Point of Care Testing for HIV Anti-Retroviral Treatment Initiation and Monitoring from Multiple or Single Fingersticks

**DOI:** 10.1371/journal.pone.0085265

**Published:** 2013-12-20

**Authors:** Natasha Gous, Lesley Scott, Joachim Potgieter, Lumka Ntabeni, Sharon Enslin, Ronel Newman, Wendy Stevens

**Affiliations:** 1 Department of Molecular Medicine and Haematology, School of Pathology, Faculty of Health Sciences, University of Witwatersrand, Johannesburg, South Africa; 2 Department of Haematology, Tshwane Academic Division and University of Pretoria, Pretoria, South Africa; 3 National Health Laboratory Service, Johannesburg, South Africa; Fundacion Huesped, Argentina

## Abstract

**Background:**

Point of Care testing (POCT) provides on-site, rapid, accessible results. With current South African anti-retroviral treatment guidelines, up to 4 fingersticks /patient/clinic visit could be required if utilizing POC. We determined the feasibility and accuracy of a nurse performing multiple POCT on multiple fingersticks followed by simplification of the process by performance of multiple POC on a single fingerstick.

**Method and Findings:**

Random HIV positive adult patients presenting at a HIV treatment clinic in South Africa, for ART initiation/ monitoring, were approached to participate in the study between April-June 2012. Phase I: n=150 patients approached for multiple POCT on multiple fingersticks. Phase II: n=150 patients approached for multiple POCT on a single fingerstick. The following POC tests were performed by a dedicated nurse: PIMA (CD4), HemoCue (hemoglobin), Reflotron (alanine aminotransferase, creatinine). A venepuncture specimen was taken for predicate laboratory methodology. Normal laboratory ranges and Royal College of Pathologists Australasia (RCPA) allowable differences were used as guidelines for comparison. In 67% of participants, ≥3 tests were requested per visit. All POCT were accurate but ranged in variability. Phase I: Hemoglobin was accurate (3.2%CV) while CD4, alanine aminotransferase and creatinine showed increased variability (16.3%CV; 9.3%CV; 12.9%CV respectively). PIMA generated a misclassification of 12.4%. Phase II: Hemoglobin, alanine aminotransferase and creatinine showed good accuracy (3.2%CV, 8.7%CV, 6.4%CV respectively) with increased variability on CD4 (12.4%CV) but low clinical misclassification (4.1%). No trends were observed for the sequence in which POC was performed on a single fingerstick. Overall, PIMA CD4 generated the highest error rate (16-19%).

**Conclusions:**

Multiple POCT for ART initiation and/or monitoring can be performed practically by a dedicated nurse on multiple fingersticks. The process is as accurate as predicate methodology and can be simplified using a single fingerstick.

## Introduction

The most important determinants of success in anti-retroviral treatment (ART) programs in South Africa rely on rapid HIV diagnosis, linkage to care, timely treatment initiation, and long-term retention of patients in care [1]. By 2012, South Africa had scaled up its ART services to access approximately 7.1 million people [2] and has 2.5 million people currently receiving treatment [3]. This rapid scale up has led to large investment in developing laboratory capacity in the public sector through the expansion of the National Health Laboratory Services (NHLS) centralised laboratory infrastructure (currently 62 CD4 and 17 HIV viral load centralised laboratories). According to the Department of Health’s National Strategic Plan for 2012/2013–2016/2017, emphasis will be placed on the need for universal annual screening of HIV and TB, thus further increasing testing requirements [4]. Many HIV infected patients who need access to laboratory services for management, live in remote areas with limited access to even basic healthcare services [5]. To meet these demands, decentralisation of laboratory testing through the implementation of Point-of-Care (POC) may provide a solution particularly, for those clinics that are low volume sites and are serviced by laboratories more than a few hours drive from the clinic. The vast numbers of patients in South Africa requiring ART initiation and monitoring however, increases the volumes of tests required, thus challenges of the feasibility of wide-scale implementation of multiple POC assays. 

POC or near-patient testing, employs small, simple-to-use, portable technologies for low volume settings [6] and are designed to allow rapid pathological sample analysis at the point of care [7], on easily available specimens such as fingerstick blood (capillary) or sputum. Fingerstick blood collection for POC testing has advantages over venous blood draw in that it is less invasive, faster to perform and provides results immediately [8]. In remote settings, phlebotomy skills are also a limiting factor to improving access to laboratory tests. In this context, the use of fingersticks and heel pricks has gained momentum in two scenarios: 1) HIV rapid diagnostic testing for adults and older children performed by lay counsellors, 2) collection of blood by heel sticks for dried blood spots, to be processed in central laboratories, for HIV exposed infants for HIV PCR assays [9,10]. 

In South Africa, the National Department of Health (NDoH) is calling for the strengthening of primary healthcare through systems re-engineering [11]. POC testing as an extension of laboratory systems and services may have a place in this process [12]. Advances in the POC testing arena may help to alleviate many of the problems faced by low resource, high HIV and TB burden settings, by providing on-site, rapid accessibility to laboratory tests and timely treatment initiation. The use of POC testing devices has previously proven feasible and accurate on fingerstick blood [13] and CD4 at POC has been shown to reduce pre-treatment patient loss to follow up and improve overall ART initiations [13-16]. 

According to the South African treatment guidelines at the time of the study [17] the initial laboratory tests needed for initiation of ART included a CD4, followed by creatinine (Cr), alanine aminotransferase (ALT) and hemoglobin (Hb). Indeed, the use of multiple POC testing platforms for this group of patients may prove beneficial, especially in terms of improving turnaround times to clinical decision-making and decreasing loss to follow up. In the scenario of a patient initiating ART as per current in-country treatment guidelines, this could require up to 4 fingersticks per visit if utilising POC testing, over and above the initial two fingersticks needed for HIV counselling and testing. An obvious way of overcoming the multiple fingerstick hurdle, would be to perform POC testing on a venepuncture specimen. This would have cost implications for added materials and require the cadre of POC staff to be trained phlebotomists. One research study has shown acceptable performance of multiple POC (up to three POC tests) for ART initiation [13], but little is known about the feasibility and accuracy of performing multiple POC testing on multiple fingersticks on a single patient and no data is available on whether this process can be simplified by performing multiple POC testing from a single fingerstick specimen. This study therefore investigated the following issues: (i) Can multiple POC testing be performed from multiple fingersticks in terms of operational practicality and still yield accurate results?; (ii) Can multiple POC testing be simplified by performing all POC testing from a single fingerstick specimen?; and (iii) Does the sequence of POC tests performed contribute to result variability? 

## Methods

### Ethics statement

The study was approved by the Faculties of Health Sciences ethics committees at both the University of the Witwatersrand, (protocol number M120143) and the University of Pretoria/Tshwane (CD4 and Hb POC protocol number 151/2010; chemistry POC protocol number 240/2010).

### Patient enrolment

Patients visiting the Comprehensive Care Management and Treatment (CCMT) clinic, Tshwane District Hospital in Pretoria, South Africa, were consented (written consent) and enrolled into the study between the periods of April to June 2012, by two trained study nurses. Criteria for inclusion in the study included: individuals >18 years of age, with known HIV positive status and presenting for either ART initiation or monitoring at designated time points. 

### Study procedures

The doctor requested the test repertoire as per National ART treatment guidelines at the time of the study [17]. The study staff collected all fingerstick specimens and performed POC testing in a designated POC testing room in the clinic. POC results were not acted on for clinical management. The POC instruments available for the study were the PIMA (Alere Inc., Waltham, MA, USA) for CD4, the HemoCue 201+ (HemoCue AB, Ängelholm, Sweden) for Hb and the Reflotron®Plus (Roche Diagnostics, GmbH, Germany) for ALT and Cr (age and sex can be used to calculate Cr clearance). These were selected based on current testing guidelines for HIV initiation and monitoring as well as instrument availability at the time of the study. An additional EDTA tube (for CD4 and Hb) and/or clotted blood (for ALT and Cr) was collected by venepuncture for predicate laboratory testing as per routine standard-of-care (SOC) and used for clinical decision-making. All blood specimens were transported to the laboratory and tested within 6 hours post-venepuncture. The NHLS laboratories performing the predicate testing all comply to Good Laboratory Practice standards and are SANAS accredited (South African National Accreditation system) [18]. 

Quality control (QC) on all the POC instruments was performed as per manufacturer’s instructions using supplier recommended material and briefly described: PIMA - daily QC with a low and high control cartridge (Alere Inc.); HemoCue – weekly QC with 3 Hemotrol controls namely, low, normal and high (Eurotrol); Reflotron – weekly QC with a universal control, Precinorm U, for Cr and ALT (Roche Diagnostics). A log sheet was used to record QC values. 

### Multiple POC testing from multiple fingerstick specimens

According to the study objectives, two phases were carried out. 

Phase 1, performance of multiple POC testing on multiple fingersticks: POC nurses recruited 150 patients into the multiple fingerstick phase of the study. This phase followed each POC test manufacturer’s standard operating procedures for blood collection by fingerstick. Each POC test was performed on a separate fingerstick using supplier recommended lancets (Table 1) [19-21]. 

**Table 1:** Supplier recommended lancets for multiple POC arm based on supplier information/recommendations [19-22].

**POC instruments**	**Supplier recommended lancets**	**Lancet Specifications**
**PIMA**	Sarstedt safety lancet	1.6mm depth
**HemoCue**	HemoCue safety lancet	2.25mm depth
**Reflotron**	Roche AccuChek Softclix Pro lancet	1.7mm depth

### Multiple POC testing from a single fingerstick specimen

Following on the first phase, phase 2 measured the performance of multiple POC tests on a single fingerstick. 150 patients were enrolled into this phase, which followed a simplified version of the manufacturer’s standard operating procedures for each POC test; if multiple POC tests were requested, all tests were performed on a single fingerstick (depending on the amount of blood available) using a single lancet. The lancet chosen for this arm was the PIMA Sarstedt safety lancet (Sarstedt Group), as it uses a blade to produce a finger slice as opposed to a traditional fingerstick and thus produces a larger amount of blood [22]. If insufficient blood was available to perform all the tests requested on the single fingerstick, a second fingerstick was performed. 

### The sequence of POC tests from a single fingerstick

The sequence of blood collection and testing was changed during the second phase of the study to allow for the different instruments to be tested first on the single fingerstick (n = 50 for each sequence). Sequence 1: Reflotron followed by HemoCue and PIMA; Sequence 2: HemoCue, Reflotron, PIMA; Sequence 3: PIMA, Reflotron then HemoCue. 

Any POC instrument/cartridge errors/failures in either phase were only repeated with an additional fingerstick if the patient was willing. 

### Predicate laboratory testing procedures

The predicate methodology used by the Core Laboratory included: PLG CD4 using the FC 500 (Beckman Coulter, Miami, FL); Hb using Advia 120 and 2120 analysers (Siemens Diagnostic Solutions, Tarrytown, NY); ALT and Cr on the Synchron DXC 800 (Beckman Coulter, Miami, FL). The normal ranges for each analyte using predicate methodologies were used as a reference for determining potential clinical changes in decision-making if POC results had been used. These reference ranges were: Hb 12g/dL - 18g/dL; ALT 10-40U/l; Cr 64-104umol/l; a cut off of 350 cells/ul for CD4 was applied for clinical misclassification. The Royal College of Pathologists of Australasia (RCPA) allowable differences [23] were also used as guidelines for assessing performance of each of the phases, these were: Hb ±0.5<10g/dL and ±5%>10g/dL, ALT ±5≤40 U/l and ±12%>40U/l, Cr±8<100umol/l and ±8%≥100umol/l.

Comparison was also made in terms of the sequence in which analytes were tested on POC instruments from a single fingerstick (phase 2). 

### Statistical analysis

The numbers of tests (fingersticks required) per patient and test (or instrument) errors per phase were quantified. T-tests were used to determine any difference in age and CD4 count and a Chi-squared test was used to determine any difference in gender between the two groups with 95% confidence. Assay performance (precision and accuracy) and method comparison (agreement) between POC and predicate methodology was measured using mean, median, range, percentage similarity (using percentage similarity standard deviation [SD] and coefficient of variation [7] [24]) and Bland-Altman (using bias) [25]. Functions were performed using STATA 12. Scatter plots were used to represent outliers and included the normal reference ranges. Similar scatter plots were used to visualize sequence of testing during the single fingerstick phase. Misclassification for CD4 was determined using the 350 cell/ul threshold and sensitivity and specificity were calculated.

## Results

### Patient demographics

**Figure 1:**
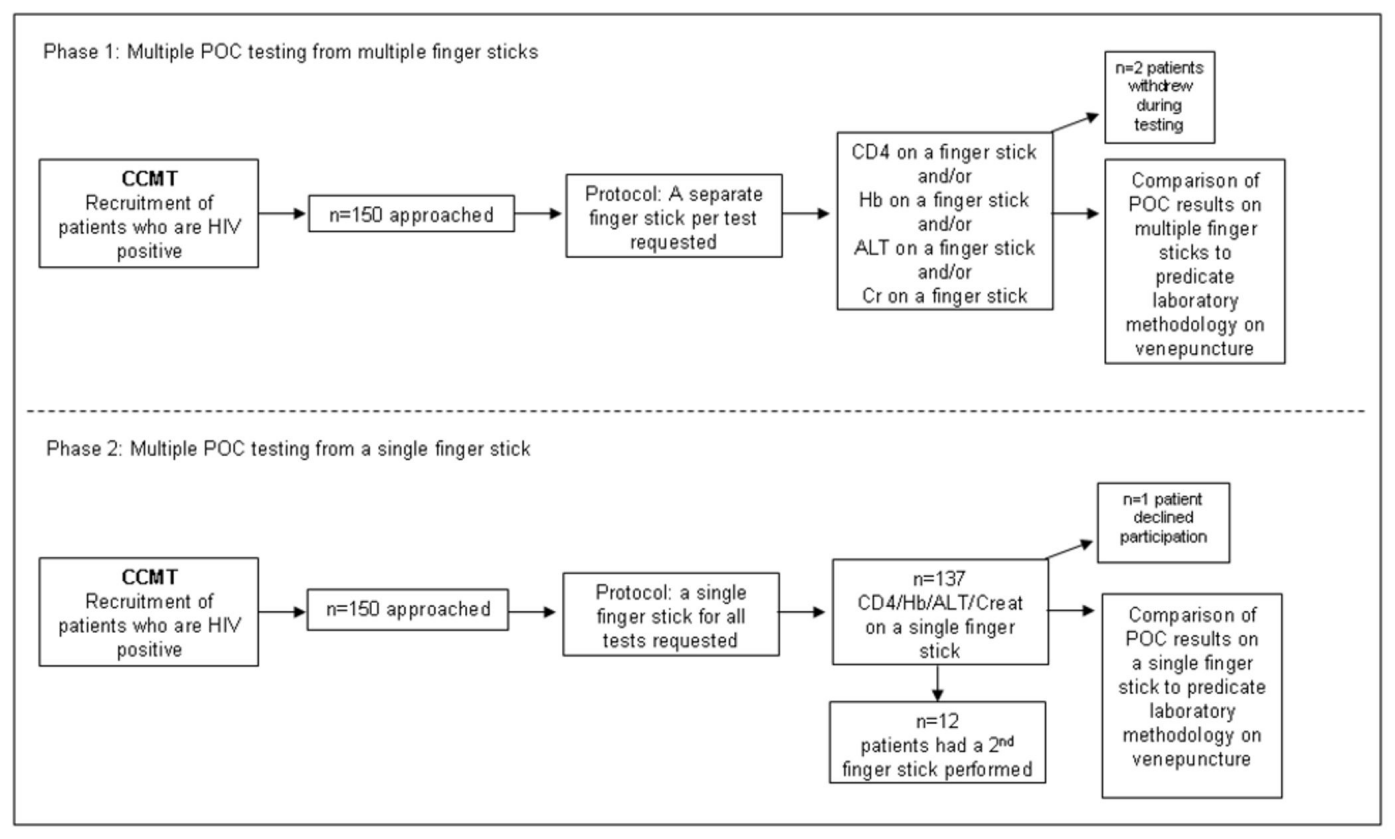
Schematic of study design.

The mean age of all participants consented and enrolled between the 30^th^ April and 15^th^ June 2012 was 35.5 years (n=299) of which 75.6% were female (n=226). There was no significant difference in the two groups (multiple or single fingerstick) for patient age (p=0.64), gender (p=0.24) or CD4 count (p=0.65). Of the total patients enrolled into the study, 67% required three or more tests per single visit based on standard ART guidelines at time of study (South African 2010 guidelines). A schematic of the study design is shown in Figure 1. 

### Multiple POC testing from multiple fingerstick specimens

One hundred and fifty patients were approached for this first part of the study. Two patients, both requiring four POC tests withdrew from the study; one after receiving two fingersticks and the other after receiving three fingersticks (Figure 1). Six percent (9/150) of patients required a 5^th^ or 6^th^ fingerstick to complete the POC test repertoire, mostly for CD4 and Cr due to poor blood flow from the fingerstick. The total number of tests requested during phase 1 was 475 with Cr the most frequently requested analyte. The PIMA instrument reported the highest error rate at 16.33% (n=16) (7 exposure control, 3 cell movement control, 1 reagent control, 4 image control, 1 gating control error), of which 6 were repeated on another fingerstick. The Reflotron had 2 operator errors and 4 POC tests could not be completed due to insufficient blood flow. Three venepuncture samples were rejected from laboratory testing during this phase. 

**Figure 2:**
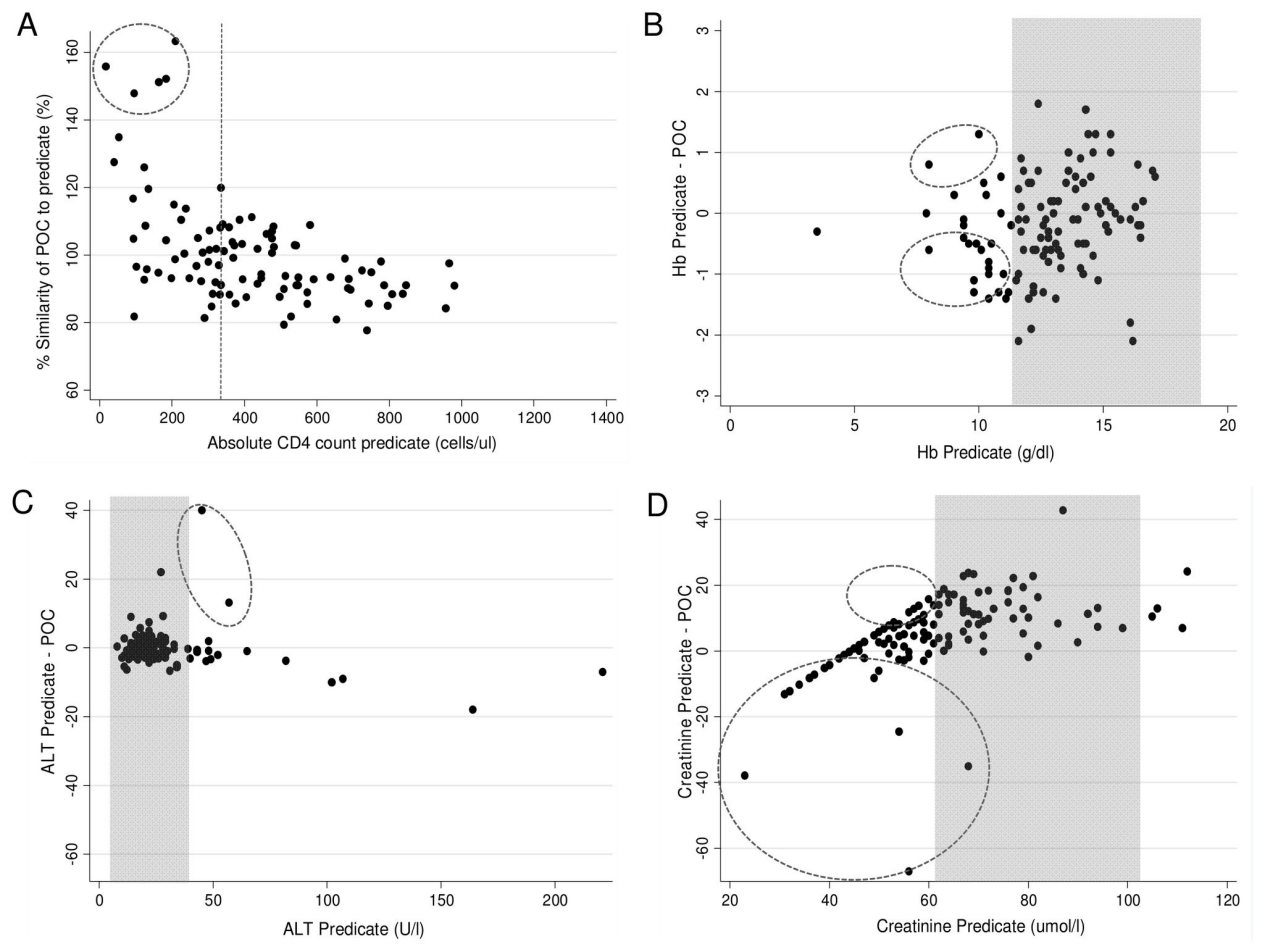
Scatter plots of method comparison for POC testing from multiple fingersticks compared to routine laboratory methodology (Phase I). The vertical axis in each plot is either percentage similarity (A) or difference (B-D) between POC and predicate and the horizontal axis is the absolute value of predicate tests: A) Percentage similarity for CD4. Red dashed line indicates misclassification point of 350 cells/ul and circle highlights outliers. B-D) Bland Altman difference scatter plots for POC versus predicate methodology for Hb (B), ALT (C) and Cr (D). Grey areas indicate normal ranges for analytes based on predicate method and circles highlight outliers mostly based on RCPA guidelines.

Method comparison of the POC test results on multiple fingerstick specimens versus predicate laboratory methodology is shown in Table 2 and Figure 2A-D. PIMA CD4 demonstrated good accuracy (mean percentage similarity of μ=100.7%) but increased variability (percentage similarity SD 16.5%), compared to the laboratory predicate assay. Bias was acceptable (32 cells/ul) for the data set (~median of 380 cells/ul). The scatter plot in Figure 2A demonstrated a downward trend in PIMA CD4 results indicating that the PIMA reads higher in the low CD4 count (<350 cells/ul) range and lower in the high CD4 count (>500 cells/ul) range, compared to the predicate laboratory method. A few outliers are visible, but all are below the <350 cells/ul category. The overall misclassification of PIMA CD4 at the 350 cells/ul threshold was 12.4% giving a sensitivity of 86.4% (Table 3). 

**Table 2:** Method comparison of POC results for all analytes versus predicate laboratory methodology.

**Variables**	**CD4 (cells/ul)**	**Hb (g/dl)**	**ALT (U/l)**	**Cr (umol/l)**
**Phase 1: Multiple fingersticks vs predicate**				
**N**	98	115	115	131
**Mean (range)**	384 (36-917)	12.9 (3.8-18.3)	35.3 (5-775)	59.6 (44.2-528)
**Median**	380	13.1	22	52.1
Bias* (95% CI)	32 (12; 53)	-0.19 (-0.34 ;-0.05)	-0.58 (-2.11;0.95)	5.32 (3.14;7.5)
**Bias SD**	101.33	0.8	8.3	12.6
**Mean % similarity**	100.7	100.9	100	97.4
**% Similarity SD**	16.5	3.2	9.3	12.6
**% Similarity CV**	16.3	3.2	9.3	12.9
**Phase 2: Single fingerstick vs predicate**				
**N**	73	94	97	129
**Mean (range)**	402 (42-824)	12.5 (6.1-17.1)	27.8 (5-165)	53.8 (44.2-97)
**Median**	398	12.5	21	49.4
Bias* (95% CI)	30 (-3; 63)	-0.3 (-0.49 ;-0.19)	0.64 (-0.69;1.97)	4.95 (3.68;6.22)
**Bias SD**	143.7	0.7	6.6	7.3
**Mean % similarity**	99.4	101.5	99.1	96.4
**% Similarity SD**	12.4	3.2	8.6	6.2
**% Similarity CV**	12.4	3.2	8.7	6.4

**Table 3:** ART eligibility misclassification, sensitivity and specificity of PIMA based on a CD4 threshold of 350 cells/ul for phase I (multiple fingerstickS) and II (single fingerstick).

	**True Positive**	**False Positive**	**True Negative**	**False Negative**	**Total misclassi-fication**	***Sensitivity at 350 cells/ul (95% CI)***	***Specificity at 350 cells/ul (95% CI)***
**Multiple (n=96)**	38 (39.6%)	6 (6.2%)	46 (48%)	6 (6.2%)	12.4%	86.4% (72; 94)	88.5% (76; 95)
**Single (n=73)**	30 (41.1%)	2 (2.7%)	40 (54.8%)	1 (1.4%)	4.1%	97.5% (81.4; 99.8)	95% (82.5; 99.1)

HemoCue Hb showed good accuracy (mean percentage similarity of 100.9%), precision and overall agreement (percentage similarity SD and %CV of 3.2). Figure 2B shows random scatter of outliers (16.5%, 19/115) based strictly on the RCPA guidelines. As the bias is low (-0.19) compared to predicate methodology, it would not alter clinical decision-making.

ALT testing on the Reflotron was 100% accurate for phase I when compared to predicate method and showed good precision (9.3%) and overall agreement (percentage similarity CV 9.3%). Random scatter of values across the data set with very few outliers are visible (1.7%, 2/115) using RCPA allowable differences (Figure 2C). As both these outliers were above the normal predicate reference range of 40U/l, they may have affected clinical decision-making. The mean negative bias of -0.58U/L was low.

The mean percentage similarity for Cr measurements generated lower values than predicate methodology (97.4%) and showed variability (percentage similarity of SD12.6%) attributable to outliers (19.1%, 25/131). The scatter plot (Figure 2D) also shows a trend due to the minimum cut off of 44.2umol/l on the Reflotron instrument, as well as a trend towards reading lower than predicate methodology as Cr levels increase. However, an overall acceptable bias (within RCPA limits) was observed.

### Multiple POC testing from a single fingerstick specimen

One hundred and forty nine patients where consented for this part of the study, with one individual declining to participate. The total number of tests requested was 407 with Cr again the most frequently tested analyte. All the POC tests requested could be completed on 91.9% (137/149) of patients from a single fingerstick specimen and 8.1% (12/149) required a second fingerstick (Figure 1). PIMA generated the highest error rate of 19.18% (n=14) (9 cell movement control, 1 insufficient volume, 3 exposure control, 1 gating control error), of which only one could be repeated on a second fingerstick specimen. One CD4 POC test could not be completed due to insufficient blood flow. No errors were observed from any other POC instruments.

**Figure 3:**
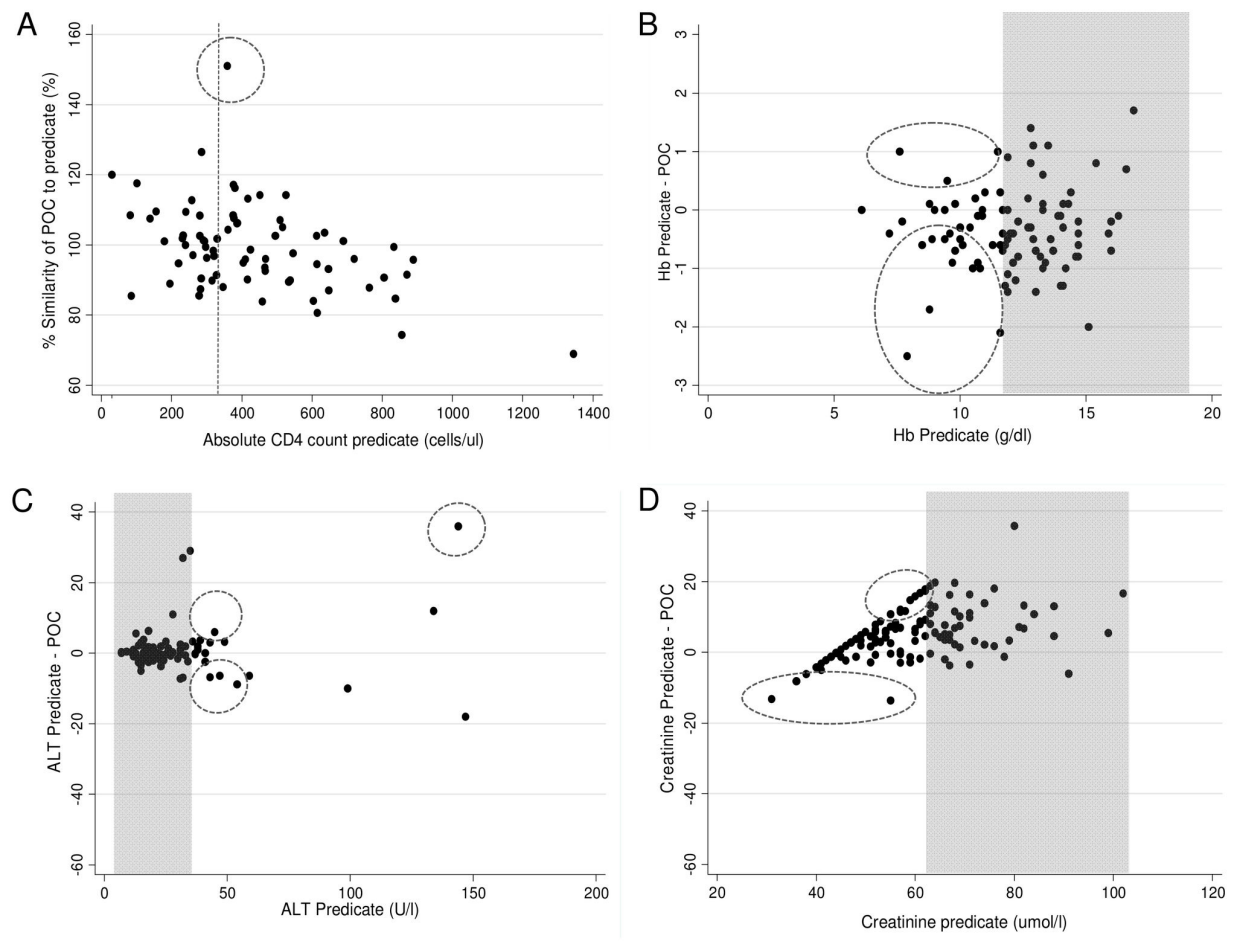
Scatter plots of method comparison of POC testing from a single fingerstick compared to routine laboratory methodology (phase II). A) Percentage similarity for CD4. Red dashed line indicates misclassification point of 350 cells/ul and circle highlights outliers. B-D) Bland Altman difference scatter plots for POC versus predicate methodology for Hb (B), ALT (C) and Cr (D). Grey areas indicate normal ranges for analytes based on predicate method and circles highlight outliers mostly based on RCPA guidelines.

Method comparison of analyte results from phase II are reported in Table 2 and Figure 3A-D. From a single fingerstick, PIMA CD4 demonstrated good accuracy (mean percentage similarity μ=99.4%) and a bias of 30 cells/ul (median of 398 cells/ul) but increased variability of 12.4% percentage similarity SD. One outlier is visible above 350 cells/ul, but would not have changed clinical patient management as both PIMA and predicate technologies identified this patient as not suitable for ART initiation. The overall misclassification of PIMA CD4 at the 350 cells/ul threshold was 4.1%, giving a sensitivity of 97% (Table 3). 

Hb results also showed good accuracy (mean percentage similarity of 101.5%), precision and overall agreement (percentage similarity SD and %CV of 3.2) and low bias compared to predicate, however applying strict RCPA limits, 20.2% (19/94) of results would be considered outliers (Figure 3B).

ALT measurements from a single fingerstick showed good accuracy and precision (99.1% and 8.6% respectively) and similar overall agreement (percentage similarity CV 8.7%). A few random outliers (using RCPA limits) are highlighted in Figure 3C (5.2%, 5/97), all above 40U/l, which potentially could have affected clinical decision-making. Single fingerstick testing showed a positive but low bias that was different to the negative but low bias in multiple fingerstick testing. 

Cr testing generated lower values than predicate methodology (μ=96.4%) with low variability (percentage similarity SD6.2%) and bias of 4.95umol/l (within RCPA limits). A trend however, is present in Figure 3D with Reflotron generating lower values as Cr levels increase, resulting in potentially 19 outliers (14.7%).

### Sequence analysis of multiple POC testing on a single fingerstick

**Figure 4:**
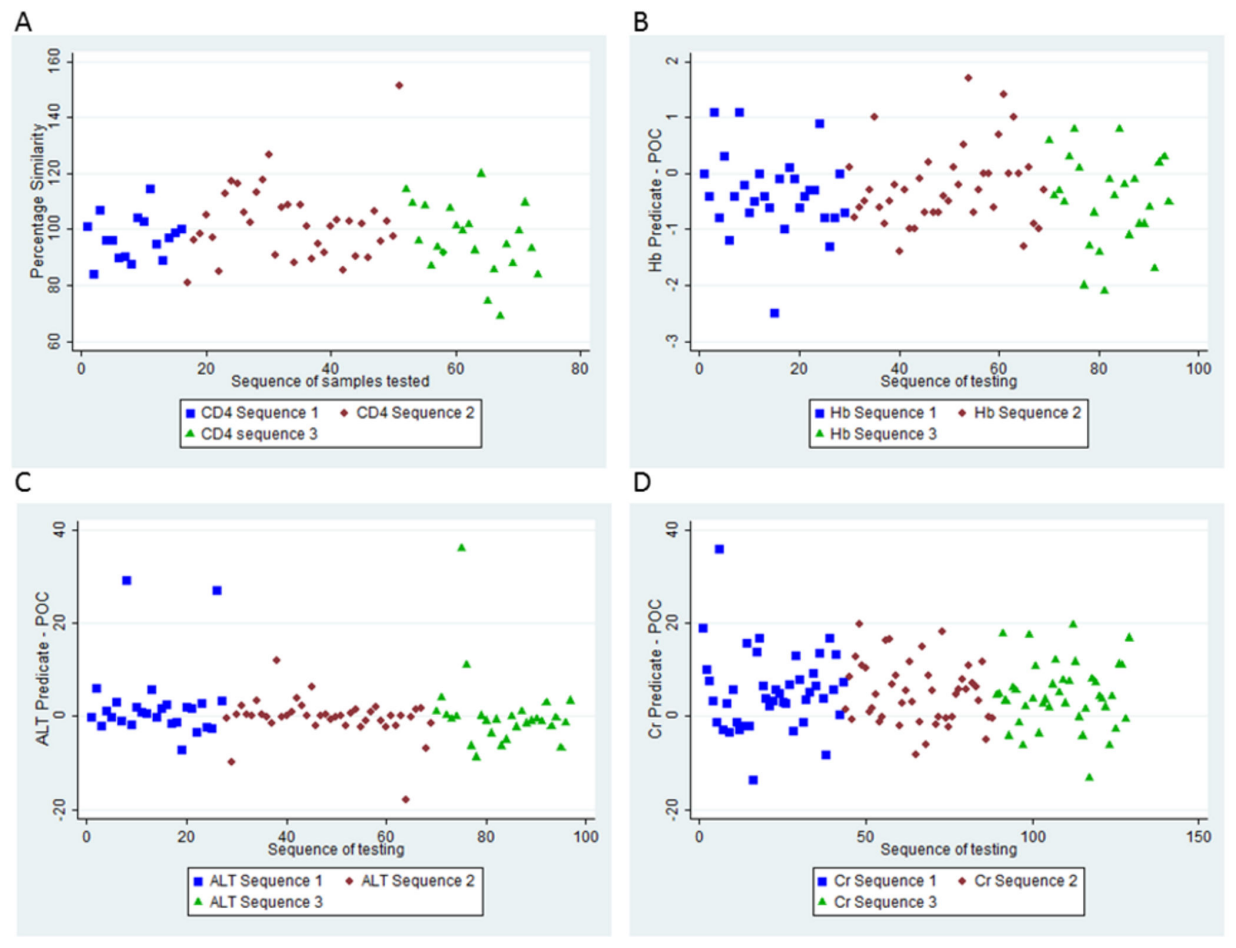
Scatter plots showing sequence of POC testing from a single fingerstick (phase II) compared to routine laboratory methodology. A) Percentage similarity for CD4. B-D) Bland Altman difference scatter plots for sequence of POC testing versus predicate methodology for Hb (B), ALT (C) and Cr (D). Blue indicates sequence 1: Reflotron followed by HemoCue and PIMA; Red indicates Sequence 2: HemoCue, Reflotron, PIMA; and green indicates Sequence 3: PIMA, Reflotron then HemoCue.

Evaluation of the POC test results across the sequence of testing from a single fingerstick specimen showed random distribution of outliers and no trends were evident across assay performance (Figure 4A-D).

### Quality control

The quality control material on all the POC instruments performed within acceptable limits, with only one outlier occurring on the PIMA low control. After repeat testing, this control performed within acceptable manufacturer’s standards.

## Discussion

Many potential benefits are reported after introduction of POC testing into non-laboratory environments in low resource countries, such as reduced skill requirements, faster turnaround times, better patient management and resource utilization [6] and improved staff satisfaction [26]. In South Africa, which has the largest ART roll out program in the world, the implementation of multiple POC testing for monitoring could be highly beneficial for detecting acute and chronic adverse events associated with ART [27]. It has been proposed that POC should be an extension of the laboratory network using the tiered approach, as proposed by the Maputo declaration [28]. However, this small study highlights that more than half of patients attending the clinic for routine ART monitoring/initiation, required 3 to 4 tests per visit. This equates to multiple fingersticks per patient, per visit if POC is introduced. The operational capacity and feasibility of this practice on a national scale needs further analysis. Study findings demonstrated that multiple POC tests performed as per manufacturer’s instructions (i.e. single fingerstick using supplier recommended lancet) can practically be performed by a dedicated nurse. Only 6% of subjects needed a 5^th^ or 6^th^ fingerstick to complete the POC test repertoire in this cohort. However in more remote settings where individuals undertake manual labour such as in farming or mining communities, obtaining a fingerstick specimen from calloused fingers may be more difficult. This is being investigated in a further study. Overall, the performance of the POC analytes, compared to predicate laboratory methodology, was accurate for Hb and ALT. Increased variability was more evident with PIMA CD4 and Reflotron Cr. 

In an attempt to simplify the POC testing process from necessitating multiple fingersticks and reduce discomfort to patients, we performed multiple POC tests on a single fingerstick specimen. This process was found to be practical and simple and only a small percentage of patients (8%,12/149) required a second fingerstick to complete the requested POC testing repertoire. Advantages of this simplified testing would be reduction in the number of fingersticks per patient and thus a significant reduction in nurse exposure to blood and other contaminants, reduced discomfort to the patient, as well as a reduction in consumables used. The overall performance of the various POC tests from a single fingerstick was acceptable for all analytes tested compared to predicate methodology. Based on the RCPA guidelines, some values may be considered outliers on the plots for Hb, ALT and Cr, however no trends or differences were visible between the sequence in testing protocol from a single fingerstick.

In scrutinizing the performance of each platform in more detail, starting with the CD4 test, examples of within technology variability such as FACSCount and PLG studies report acceptable variability (%CV) on repeat venous sample testing of 5%-9.3% [29] [30]. PIMA within variability (and across 5 different instruments) on venous samples (range in CD4 of 44 -504 cells/ul) shows higher but acceptable variability ranging from 4.2% to 15.5% respectively [31].

The PIMA CD4 evaluation in our study (across technology) on fingerstick specimens generated values at this upper venous limit (16.3%CV for phase 1 and 12.4%CV for phase 2), reflecting the PIMA acceptability for ART initiation, but less assurance from this study data if this technology were to be used for monitoring (unless venepuncture specimens are tested). This latter application however, is of less concern with the new South African and WHO ART 2013 guidelines using CD4 for initiation and a reduction in CD4 monitoring after the first year, with greater emphasis on VL monitoring. The alternative would be to use venepuncture for PIMA CD4 monitoring. Increases in variability of capillary PIMA CD4 testing have previously been documented, where % similarity CV’s ranged from 11% in a Johannesburg Clinic, to as high as 28.8% in an antenatal hospital clinic [32]. Irrespective of this variability, the overall bias in PIMA CD4 versus predicate testing in both the phases is acceptable (~30 cells/ul) for the range in CD4 count (median >350 cells/ul), however the variability (SD) of this bias was broad (~100 cells) probably indicative of a small sample size. The total misclassification of 12.4% for multiple fingersticks and 4.1% for testing on a single fingerstick has similarly been documented in other studies and shows more patients would be initiated on ART than missed if testing were done on a single fingerstick specimen [33-35]. Concern is noted over the high PIMA CD4 error rate (16% and 19%), which was higher than any other POC instrument used in our study, and was slightly higher during the single fingerstick phase. Similar error rates for PIMA CD4 have been observed in other reports [32,34]. 

The HemoCue instrument’s good performance for Hb measurements on fingerstick, whether multiple or single (bias of -0.2±0.8g/dL multiple and -0.4±0.9g/dL for single) is in contrast to literature which demonstrates significant variability in capillary blood measurements [36] and a tendency for POC to increase at higher Hb values [37,38]. Bland Altman difference plots showed random distribution of outliers but more outliers (16% versus 20%) are visible when POC was performed from a single fingerstick specimen. 

Reflotron performed well for ALT measurements, regardless of whether single or multiple fingersticks were performed. These findings are in contrast to Grounden et al [27] who found significant biases for Reflotron ALT. The better performance in our study for both phases could be attributed to the experience and thorough training of the nurse operators that may have resulted in better sample collection. With creatinine measurements however, which are known to be challenging due to haematocrit variations and interference by bilirubin [39], the Reflotron was found to read lower than predicate method as creatinine levels increased, regardless of whether POC was performed from multiple or a single fingerstick. Similar findings have been observed in other studies where Reflotron was found to underestimate creatinine measurements at concentrations between 90 and 150umol/L [39]. One factor that may result in overestimation of creatinine in the routine laboratory specimens is sample haemolysis [40] which is mainly due to poor sample collection. However, we do not believe this to have influenced our results, as these samples are generally checked and rejected by the routine laboratory. 

In summary, this study demonstrates the feasibility of performing multiple POC testing on multiple fingersticks to accurately monitor ARV treatment. We also demonstrated that a single fingerstick produces sufficient blood to accurately perform up to four POC tests (approximately 95ul of blood), to simplify the testing process. This may be the preferred method to ensure quality testing if multiple POC tests are to be introduced for ART initiation in South Africa. 

POC testing can reliably and accurately be performed on fingerstick blood thereby minimizing potential bio-hazardous risk introduced by uncapping EDTA tubes and pipetting of venepuncture blood, does not require a trained phlebotomist, generates minimal biological waste, is minimally invasive and relatively easy to perform [41] but multiple POC will need dedicated staff. Patient acceptance of multiple fingersticks for POC testing is also a consideration for uptake of POC and is being evaluated in a further study. We envisage that a new cadre of staff would need to be trained for POC operation, one that has both technical skills and clinical knowledge. Monitoring of quality POC testing will have to be a component of implementation, as previously outlined for these issues around HIV rapid testing [42]. 

Limited guidelines on multiple POC testing for ART initiation and monitoring are available, so field testing studies such as this are important to understanding how POC performs in the field. Many other obstacles to implementation will need clarification before POC can be implemented. Depending on where POC is placed, different facilities will likely have differing needs and resources; throughput of POC instruments will have to be taken into consideration; management of stock control and quality control; the cadre of POC staff needed; management of testing volumes and results; impact on patient care. 
